# Supplementary material for: Prevalence and risk factors of cutaneous leishmaniasis in a newly identified endemic site in South-Ethiopia
Source: PLoS One. 2024 Dec 30;19(12):e0311917. doi: 10.1371/journal.pone.0311917 (PMC11684615; doi:10.1371/journal.pone.0311917)
Supplement: S1 Table — (DOCX) [file pone.0311917.s001.docx]

**Supporting Information**

**Table S1. House, environmental, and behavioral characteristics in the cutaneous leishmaniasis (CL) in the CL prevalence survey, Bilala Shay, Ethiopia 2021 (N=252).**

| **Household characteristic** | | | **N (%) or median (IQR)** |
| --- | --- | --- | --- |
| Number of household members; median (IQR) | | | 3.5 (2-6) |
| Number of rooms; median (IQR) | | | 3 (2-3) |
| Latrine available | | | 213 (85) |
| Kitchen inside the house | | | 72 (29) |
| Smoke produced in sleeping area at night | | | 218 (86) |
| Roof structure | | |  |
| Iron sheet | | 164 (65) |  |
| Grass/bamboo | | 88 (35) |  |
| Wall structure | | |  |
| Wood only | | 23 (9) |  |
| Grass only | | 76 (30) |  |
| Wood, grass and mud | | 153 (61) |  |
| House floor structure | | |  |
| Soil, mud and dung | | 252 (100) |  |
| Cracks in the house | | | 192 (76) |
| Domestic/livestock animals in house/compound | | |  |
| Goat/sheep | | 143 (57) |  |
| Chicken | | 110 (44) |  |
| Dogs/cats | | 55 (22) |  |
| Mules/donkeys | | 7 (2.5) |  |
| Animal dung near the house | | | 194 (77) |
| Small animal burrow near the house | | | 221 (88) |
| Stone fence around the house | | | 21 (8) |
| Owning farmland near hyrax habitat | | | 156 (62) |
| Gorge < 300 meter of the house | | | 112 (44) |
| Hyrax habitat within 300 meters | | | 146 (58) |
| **Behavioural characteristic** | | |  |
| Spending time outside during late evening | | | 472 (46.6) |
| Activities outside during late evening | | |  |
| Not going out | 540 (53.4) |  |  |
| Playing | 32 (3.2) |  |  |
| Fetching water/fire wood | 181 (17.9) |  |  |
| Herding animals | 98 (9.7) |  |  |
| Farming work | 161 (15.9) |  |  |
| Spending evening outside where hyraxes reside | | | 480 (47.4) |
| Sleeping under a bed net | | | 27 (2.7) |

IQR: interquartile range
