# Supplementary material for: Prevalence and risk factors of cutaneous leishmaniasis in a newly identified endemic site in South-Ethiopia
Source: PLoS One. 2024 Dec 30;19(12):e0311917. doi: 10.1371/journal.pone.0311917 (PMC11684615; doi:10.1371/journal.pone.0311917)
Supplement: S2 Table — (DOCX) [file pone.0311917.s002.docx]

**Supporting Information**

**Table S2. Behavioral risk factors by age and sex in the cutaneous leishmaniasis prevalence survey, Bilala Shay, Ethiopia 2021 (N=1012).**

| **Behavioral RFs** | **Males; n (column %)** | | | | | **Females; n (column %)** | | | | |
| --- | --- | --- | --- | --- | --- | --- | --- | --- | --- | --- |
| **Activity outside during the late evening** | **< 5 years** | **5-11 years** | **12-17 years** | **> 18 years** | **< 5 years** | | **5-11 years** | **12-17 years** | **> 18 years** |  |
| None | 34 (94) | 49 (56) | 44 (52) | 120 (41) | 31 (100) | | 46 (67) | 45 (51) | 171 (53) |  |
| Playing | 1 (3) | 7 (8) | 5 (6) | 13 (4) | 0 (0) | | 4 (6) | 1 (1) | 1 (0.3) |  |
| Fetching water/firewood | 0 (0) | 8 (9) | 2 (2) | 4 (1) | 0 (0) | | 16 (23) | 37 (42) | 114 (35) |  |
| Herding animals | 1 (3) | 22 (25) | 28 (33) | 29 (10) | 0 (0) | | 3 (4) | 5 (6) | 10 (3) |  |
| Farming | 0 (0) | 2 (2) | 5 (6) | 125 (43) | 0 (0) | | 0 (0) | 0 (0) | 29 (9) |  |
| **Spending evening outside where hyraxes reside** |  |  |  |  |  | |  |  |  |  |
| No | 34 (94) | 47 (53) | 45 (54) | 121 (42) | 31 (100 | | 45 (65) | 46 (52) | 163 (50) |  |
| Yes | 2 (6) | 41 (47) | 39 (46) | 170 (58) | 0 (0) | | 24 (35) | 42 (48) | 162 (50) |  |
