# Supplementary material for: Prevalence and risk factors of cutaneous leishmaniasis in a newly identified endemic site in South-Ethiopia
Source: PLoS One. 2024 Dec 30;19(12):e0311917. doi: 10.1371/journal.pone.0311917 (PMC11684615; doi:10.1371/journal.pone.0311917)
Supplement: S3 Table — (DOCX) [file pone.0311917.s003.docx]

**Supporting Information**

**Table S3. Association between outdoor activities during late evening and spending time outside in areas where hyraxes reside in the CL prevalence survey, Bilala Shay, Ethiopia 2021 (N=1012)**

| **Activity outside during late evening** | **Spending evening outside where hyraxes reside** | |
| --- | --- | --- |
|  | **Yes** | **No** |
| None | 22 (4) | 518 (96) |
| Playing | 28 (87) | 4 (13) |
| Fetching water/firewood | 179 (99) | 2 (1) |
| Herding animals | 96 (98) | 2 (2) |
| Farming | 155 (96) | 6 (4) |
