# Supplementary material for: Prevalence and risk factors of cutaneous leishmaniasis in a newly identified endemic site in South-Ethiopia
Source: PLoS One. 2024 Dec 30;19(12):e0311917. doi: 10.1371/journal.pone.0311917 (PMC11684615; doi:10.1371/journal.pone.0311917)
Supplement: S4 Table — (DOCX) [file pone.0311917.s004.docx]

**Supporting Information**

**Table S4. Univariate association between socio-demographic and behavioural characteristics and cutaneous leishmaniasis (CL) in the CL prevalence survey, Bilala Shaye, Ethiopia 2021 (N=1012)**

| **Variable** | **Active CL/scar;**  **n (%)** | **No active CL/scar; n (%)** | **Crude OR (95% CI)** | **P value** |
| --- | --- | --- | --- | --- |
| **Age** |  |  |  | **<0.001** |
| 0-4 year | 6 (9) | 61 (91) | 1 |  |
| 5-17 year | 125 (38) | 204 (62) | 6.6 (2.6-16.5) |  |
| 18-44 year | 132 (44) | 169 (56) | 8.6 (3.4-21.4) |  |
| 45 and above | 144 (46) | 171(54) | 9.4 (3.8-23.7) |  |
| **Sex** |  |  |  | 0.41 |
| Male | 194 (39) | 305 (61) | 1.1 (0.9-1.5) |  |
| Female | 213 (41) | 300 (59) | 1 |  |
| **Occupation** |  |  |  | 0.11 |
| Farmer/daily labourer | 289 (39) | 459 (61) | 1.3 (1.0-1.7) |  |
| Other | 118 (45) | 146 (55) | 1 |  |
| **Spending time outside where hyraxes reside** |  |  |  | **<0.001** |
| No | 172 (33) | 360 (68) | 1 |  |
| Yes | 235 (49) | 245 (51) | 2.5 (1.8-3.4) |  |
| **Reasons for activities outside** |  |  |  | **<0.001** |
| Not going out | 182 (33.7) | 358 (66.3) | 1 |  |
| Playing | 6 (18.7) | 26 (81.2) | 0.54 (0.2-1.2) |  |
| Fetching water/fire wood | 90 (49.72) | 91 (50.3) | 2.3 (1.6-3.6) |  |
| Herding animals | 44 (44.9) | 54 (55.1) | 1.9 (1.1-3.2) |  |
| Farming work | 85 (52.8) | 76 (47.2) | 2.7 (1.7-4.1) |  |
| **Spending time outside during late evening** |  |  |  | **<0.001** |
| No | 225 (48) | 247 (47.7) | 1 |  |
| Yes | 182 (33.7) | 358 (66.3) | 2.1 (1.5-3.0) |  |
| **Sleeping under bednets** |  |  |  | 0.96 |
| No | 396 (40.4) | 585 (60.6) | 1 |  |
| Yes | 11 (40) | 16 (60) | 1 (0.4-2.5) |  |

OR: odds ratio; CI: confidence interval
