# Supplementary material for: Prevalence and risk factors of cutaneous leishmaniasis in a newly identified endemic site in South-Ethiopia
Source: PLoS One. 2024 Dec 30;19(12):e0311917. doi: 10.1371/journal.pone.0311917 (PMC11684615; doi:10.1371/journal.pone.0311917)
Supplement: S5 Table — (DOCX) [file pone.0311917.s005.docx]

**Supporting Information**

**Table S5. Univariate association between household features and cutaneous leishmaniasis (CL) in the CL prevalence survey, Bilala Shaye, Ethiopia 2021 (N=1012)**

| **Variable** | **Active CL/scar; n (%)** | **No active CL/ scar; n (%)** | **Crude OR (95% CI)** | **P value** |
| --- | --- | --- | --- | --- |
| **Number of rooms in the house** |  |  |  |  |
| One | 86 (36.29) | 151 (63.71) | 1 | 0.19 |
| Two | 80 (38.10) | 130 (61.9) | 1.1 (0.7-2.0) |  |
| Three or more | 241 (42.65) | 324 (57.35) | 1.3 (0.8-2.0) |  |
| **People living in the same house** |  |  |  |  |
| 1-3 | 116 (46) | 135 (54) | 1 | 0.13 |
| 4-6 | 99 (39) | 157 (61) | 0.7 (0.5-1.0) |  |
| 7-9 | 113 (40) | 173 (60) | 0.6 (0.3-1.0) |  |
| ≥ 10 | 79 (36) | 140 (64) | 0.6 (0.4-1.0) |  |
| **House Roof structure** |  |  |  |  |
| Iron sheet | 275 (42.7) | 369 (57.3) | 1 | 0.12 |
| Grass/bamboo | 132 (35.9) | 236 (64.1) | 0.7 (0.5-1.0) |  |
| **House Wall structure** |  |  |  |  |
| Wood only | 31 (43.7) | 40 (56.3) | 1 | 0.24 |
| Grass only | 252 (41.9) | 350 (58.1) | 0.7 (0.4-1.5) |  |
| Wood, mud/grass | 124 (36.6) | 215 (63.4) | 0.6 (0.3-1.3) |  |
| **House floor structure** |  |  |  |  |
| Soil, dung or mud | 407 (40.2) | 605 (59.8) | - |  |
| Other | 0 | 0 |  |  |
| **Latrine available** |  |  |  |  |
| No | 65 (43.0) | 86 (56.9) | 1 |  |
| Yes | 342 (39.7) | 519 (60.3) | 0.9 (0.6-1.5) | 0.44 |
| **Kitchen** |  |  |  |  |
| Outside the house | 100 (34.8) | 187 (65.2) | 1 | 0.13 |
| Inside the house | 307 (42.3) | 418 (57.7) | 1.3 (0.9-1.9) |  |
| **Smoke in sleeping area during night** |  |  |  |  |
| No | 54 (42) | 74 (58) | 1 |  |
| Yes | 353 (40) | 531 (60) | 1.1 (0.6-1.8) | 0.63 |
| **Presence of cracks in the house** |  |  |  |  |
| No | 82 (38) | 136 (62) | 1 |  |
| Yes | 325 (41) | 469 (59) | 1.1 (0.8-1.7) | 0.46 |
| **Domestic animals** |  |  |  |  |
| **Goat or sheep** |  |  |  |  |
| None | 120 (41) | 174 (59) | 1 | **0.037** |
| Inside the house | 120 (34) | 235 (62) | 1.7 (1.1-2.5) |  |
| On the compound | 167 (46) | 196 (54) | 1.3 (0.7-2.6) |  |
| **Mules/donkeys** |  |  |  |  |
| None | 391 (40) | 586 (60) | 1 | 0.14 |
| Inside the house | 15 (55.6) | 12 (44.4) | 2.2 (0.7-6.6) |  |
| On the compound | 1 (12.5) | 7 (87.5) | 0.2 (0.1-2.0) |  |
| **Dogs/cat** |  |  |  |  |
| None | 260 (39) | 409 (61) | 1 | 0.68 |
| Inside the house | 67 (42.4) | 91 (58) | 1.1 (0.6-1.9) |  |
| On the compound | 42 (46) | 49 (54) | 1.4 (0.7-2.6) |  |
| Outside the compound | 38 (40.4) | 56 (60) | 1.1 (0.5-1.9) |  |
| **Chicken** |  |  |  |  |
| None | 177 (36) | 314 (64) | 1 | 0.13 |
| Inside the house | 159 (43.7) | 15 (54) | 1.4 (0.9-2.0) |  |
| On the compound | 71 (45.2) | 86 (55) | 1.5 (0.9-2.5) |  |
| **Farm near hyrax habitat** |  |  |  |  |
| No | 134(39) | 209 (61) | 1 | 0.65 |
| Yes | 273 (41) | 396 (59) | 1.0 (0.8-1.5) |  |
| **Small animal burrow around the house** |  |  |  |  |
| No | 54 (40) | 82 (60) | 1 | 0.89 |
| Yes | 353 (40) | 523 (60) | 1.0 (0.6-1.7) |  |
| **Animal dung around the house** |  |  |  |  |
| No | 44 (30) | 104 (70) | 1 | **0.011** |
| Yes | 363 (42) | 501 (58) | 1.9 (1.1-3.0) |  |
| **Stone fence around house** |  |  |  |  |
| No | 370 (39.3) | 571 (60.7) | 1 | 0.067 |
| Yes | 34 (48) | 37 (52.1 | 1.8 (0.9-3.5) |  |
| **Hyrax habitat within 300 meters** |  |  |  |  |
| No | 176 (40.1) | 263 (60) | 1 |  |
| Yes | 231 (40.3) | 342 (60) | 1.0 (0.7-1.4) | 0.94 |
| -**Gorge within 300 meters (from house)** |  |  |  |  |
| No | 223 (40) | 332 (60) | 1 | 0.97 |
| Yes | 184 (40.3) | 273 (60) | 1.0 (0.7-1.4) |  |

OR: odds ratio; CI: confidence interval
