# Supplementary material for: Prevalence and risk factors of cutaneous leishmaniasis in a newly identified endemic site in South-Ethiopia
Source: PLoS One. 2024 Dec 30;19(12):e0311917. doi: 10.1371/journal.pone.0311917 (PMC11684615; doi:10.1371/journal.pone.0311917)
Supplement: Attachment — Submitted filename: Comments.pdf [file pone.0311917.s007.pdf]

Title	Prevalence and risk factors of cutaneous leishmaniasis (CL) in South-Ethiopia's newest endemic site for CL.
Short title	A new CL hotspot of cutaneous leishmaniasis in the South of Ethiopia
Ms #	PONE-D-23-30018

Comments

Editorial	What does "**newest endemic site**" or "**A new CL hotspot**" mean?<br>Does it mean the study area was not endemic for CL previously. If so, what is the evidence for that? The words "new" and "newest" are misnomers, and they should be replaced from the title and from the main body of the manuscript.
	Last sentence of the "Introduction" in page 11 begins by the phrase "Our findings aim to….".<br><br>It makes sense to change it to "Our research (This research) aims to contribute to ……"
Regressions	<u>Combining active CL and past CL lesions in defining a key outcome variable</u><br>Risk of CL was a key outcome variable assessed by combining active and past CL cases for the purpose of uni-(multi-) variate analysis using regression models. There were 382 cases with past lesions (390 according to table 2) whose median age was 32. Assuming most active lesions occur during childhood, the majority of the 382 cases with scars may have occurred 20 years ago. Clearly, active and past CL lesions were temporally distant from each other. Consequently, combining the active lesions with scars in formulating the outcome variable cannot be straightforward particularly that data is based on a proportionally larger of past CL lesions (scars).<br><br>For instance, S2 Table gives the univariate analysis with a breakdown by age, and shows that not only children 5 to 17, but also adults have a higher odds of CL risk (OR = 6.6 – 9.4). However, from Table 2, it was clear that children in the age bracket 5 to 11 had the highest prevalence of active CL (7.6%), and those under 5 had 1.5% prevalence, and children older than 11 years and adults had low prevalence rates (ranging from 1.0% to 2.0%). Therefore, it was clear that combining active and healed lesions was a mistake.<br><br><u>Domestic animals and animal dung</u><br>Even though all households had animals, animal dung was found in 77% of households. One wonders why all household did not have dung in the compounds. Furthermore, it seems plausible to assume that presence of animals and animal dung are not independent of each other. In such a scenario, the regression model cannot assume these two variables to be independent of each other.

	In the univariate analysis, the presence of goats/sheep inside houses (but not in the compound) seemed to show marginal association with the outcome variable. Given the flaws in the design/analysis, caution should be made not to over-interpret the findings. The authors tried to build on this observation as an important finding (see discussion section implying attraction of sandflies by goats/sheep; also referring to a single natural infection of a goat - citing a publication of their own).<br><br><u>Multivariate analysis</u><br>In the multiple regression section, it is interesting to note that presence of domestic animals in the households did not come out as a predictor of CL incidence whereas animal dung turned out to be a remarkably significant predictive variable (univariate analysis, S3 Table).<br><br>The steps involved in generating Table 3 and the adjustments made variables-wise is not clearly presented. The structure of the table is not self-explanatory. A detailed description of the model is lacking.
Table 1	For the section of marital status, does it make sense to re-calculate the percentages by taking the denominator as 767 (those >18 yrs)? This gives 36.5%, 49.8% and 13.7% marital status for single, married and widowed/separated respectively.
Strengths and limitations	Why would the use of molecular techniques be considered as a strength? By the same token, the total population sampling seems to be related to feasibility rather than scientific justification. Apparently, the total population of the study population is small which must have incentivized the investigators to do it all without sampling. Of course, the approach minimizes sampling error.<br><br>The limitations have been pointed out appropriately but remains to be a concern. As explained above, there is a deduction that older children and adults are at higher risk of acquiring CL. A close look at the data seems to point that in fact small children in the age bracket 5-11 are the ones at highest risk.<br><br>Because the key outcome variable was comprised of both active and healed lesions, interpretation of associations between the several presumed explanatory variables and the key outcome variable remains to be obscure, and the conclusions made are not fully substantiated.
References	1) Ref # 16 does not give list of authors.<br>2) Journal names are given either abbreviated or written in full. Consistency is advised.
Others	Editorial corrections will improve the quality of the manuscript.
