# Supplementary material for: Prevalence and risk factors of cutaneous leishmaniasis in a newly identified endemic site in South-Ethiopia
Source: PLoS One. 2024 Dec 30;19(12):e0311917. doi: 10.1371/journal.pone.0311917 (PMC11684615; doi:10.1371/journal.pone.0311917)
Supplement: Attachment — Submitted filename: Response to ReviewersBS.docx [file pone.0311917.s008.docx]

Response to Reviewer’s Comments

**Our responses are in Blue**

**Reviewer #1: Abstract**
Except for the conclusion part, it is well-written and explanatory.

Major correction
- The conclusion part of the abstract needs significant modification, which is not related to the objective, method, and result.

We have reworded the conclusion to remain closer to our findings.

Minor correct
- Method part of the abstract
-'... features were collected using standardized questionnaires. Univaria...' == "standardized" ==> it is not indicated in the method section (of the study) about the method of standardization of the tools. Better to remove it.

As per the comment “Standardized” has been removed from the Abstract.

Introduction
- Well written
- Minor correction=
- The citation has problems. For instance, the 2nd paragraph 1st statement. The indicated information is not available in the references they authors cited.

Reference was corrected with the first article that reported the annual estimates of Ethiopia.

(See page 4, line 81 of the revised version)

- On the 9th line (same paragraph), the start by describing 'Two studies' and putting three references....

Thanks for spotting this. We have corrected it (see page 4, Line 89-91 of the revised version).

The authors need to make this correction through the introduction part.
Method
Minor
Study design
- 'An exhaustive cross-sectional house-to-...'. The term 'exhaustive' is subjective. Remove it.

By “Exhaustive” we are referring a complete census of residents. We changed the wording to clarify this.

--------------------
- '... Demography and Health Survey (18); activities ...' The reference and the information are not related.

Corrected (Page 7, line 153)

Statistical analysis
- 'EpiData (version 2.2.3.187, EpiData Association, Odense, Denmark) was...': Which data was entered using 'EpiData'?

All data mentioned under ‘conduct of the survey’ were entered using EpiData. We have clarified. (Page

**Results**
Individual and household characteristics
- 'A total of 1012 individuals from 252 households were included with 100% response rate.': In the method part, it is indicated that the number of village residents were 1255. Here the total number of participants are 1012 and the response rate is 100%. How?

Yes, in fact what was given in the method was the estimate of residents in the village. However, the actual count of the residents was 1012. This has happened due to a significant number of people that were previously living in Bilala Shaye, but moved out and changed their residential location to another site in search of farming land.

We have made the correction to 1012 to keep the information consistent:

--------------------------------------
- Metal roof == Iron sheet

Thanks. Correction has been made

------------------------------
- Marital status==Caution: The proportion also included NA. (Example: 38% married =It does not mean those eligible for marriage)

Thank you, valid point. We have now excluded those aged <18 (NA category) from the denominator

- Table 2. The title: It is not only per age group, rename the table

Title is improved as suggested

- the use of 'patients' term. The study participants were not the patients. Proper use of terms

We have now avoided the use of the term “patients”.

**Discussion**
- '... However, it is a very common cultural of Gamo people living in places like this to practice...': 'places like this' this need to be explain properly to remove ambigity

Thank you and correction is made.

Major
Conduct of the survey
- '...farmland, gorges or areas where hyraxes reside, ...' The method to determine the reside of hyraxes needs to be started properly. Is it the presence of gorges or other method was used to determine the hyraxes reside? If yes, indicated it or if not better to use only 'presence of gorges' only.

We have now tried to clarify this.

- '...confirm CL by PCR in a sub-group, nine random individuals with active lesions compatible...' In this part, the authors should explain more. For instance, here are some missing information. Why only nine subjects only? Brief description about the random selection of the 9 cases. Who collected the sample? etc should have to be added.

Thanks. All 25 CL active lesion suspected (clinically highly likely) were told to come to the sample collection site. However, only nine presented for sample collection and went for a confirmatory PCR testing. This is explained in the revised version. (Page 7, line 142-144 of the revised version). We have now also added who collected the sample.

Statistical analysis

- '... Factors with a P-value < 0.05 in univariate analysis were ...': In the result part (multiple regression part) and supplement tables, I did not see the reason for considering this cut-off point (for p-value). The number of IVs were not large in number (there are only 3 variables were included). So, why the p-value<0.05 for selecting the IVs. The adjustment needs inclusion of sound variables with correcting the cutoff point for p-value. In addition, all the included variables need to be described in the table.

Although we agree there are different approaches to strategies for multivariable analysis, and each has its advocates, it is fairly common to use a cut-off of P<0.05 to select variables to be included in the multivariate analysis, particularly if there is a good number of events compared to the number of variables that will be included in univariate analysis. Nevertheless, we reran the analysis using a cut-off of P<0.25, yielding exactly the same final multivariate outcome (ie retaining the same variables as shown in Table 3), so it seems our findings are not influence by the P-value cut-off used in univariable analysis. We have now also added which variables were included in the multivariable analysis (legend Table 3). We have also clarified that a backward selection process was conducted, retaining only those variables in the model with a P-value < 0.05. These variables are shown in Table 3.

Ethical issues
- What about those participants with active CL cases? Did the research provide treatment. If yes, what was the treatment? If no, why it was not?

For those participants who were confirmed positive for CL by PCR, an advice and referral slip were given to receive the available treatment at Arba Minch General hospital (46kms far) that provides SSG for diagnosed CL cases by admission.

Results
- The last citation of 1st paragraph of the result part is Table S1.: There is mismatch while citing tables. Be careful and check again to match the description with the tables

Thank you, we have corrected this across the manuscript

- '...located within 300 meters of areas where hyraxes ...': The method part did not explain properly the data collection including the 'hyraxes reside' and the method for estimating the distance

We have tried to clarify this now;

- Table 3. The description in the method section and the table are not consistent. The authors described that the p<0.05 in bivariant analysis were included in the multivariate. However, they did not include those variables. This need to be corrected or explained in the method part properly.

Factors with a P-value <0.05 in univariate analysis (the presence of animal dung, spending time outside in the evening in areas where hyraxes reside, individual age, spending time outside during late evening and the presence of goats/sheep on the compound) were included in multivariate analysis and with a back-ward selection process we maintained factors with P-values < 0.05.

We have now added which variables were included in the multivariable analysis (legend Table 3). We have also clarified that a backward selection process was conducted, retaining only those variables in the model with a P-value < 0.05. These variables are shown in Table 3.

- In the sensitivity analysis ... : The authors need to explain the reason for doing sensitivity analysis for those less than 12 years in the method part.

Thank you for your valid comment. We have given this much thought, and have acknowledged this as a limitation. The reasoning behind our approach is the following.

- Most other studies in Ethiopia have combined CL lesions and scars as outcomes, and by following this, our findings can be compared with the findings from these studies.
- Nevertheless, we also considered using only CL lesions as outcomes, but given the fact that we only had 25, and many risk factors we wanted to explore in univariable analysis. Given the rule of thumb of having 10 outcome events per factor included in univariate analysis, we felt this analysis would be underpowered and might lead to spurious associations (<https://pubmed.ncbi.nlm.nih.gov/8970487/>). Combining CL lesions and scars, would lead to a sufficient number of events. Moreover, the presence of CL scars also indicates “exposure” to CL risk “factors”, albeit more distant in time.
- However, to assess the robustness of our findings, and minimize the effect of this “exposure distant in time”, we conducted sensitivity analysis including all active CL lesions (adults and children), and all scars in children < 12 years old. As most active CL seems to occur in the age group 5-12 years, this would indicate “exposure to CL risk factors” not too long ago. In general, our findings seemed quite robust, suggesting the main model is sufficiently reliable. Nevertheless, we agree with your comment, and it is mentioned as a limitation.
- We have now explained the reasoning for the sensitivity in more detail in the Methods section;

We described this in the revised version (page 9, Line 197-206)

Discussion
- Our findings call for national evaluations in CL risk areas to more exactly define the current epidemiology and the need of care in the country: The argument used in this and previous studies does not lead for this type of conclusion. Because (1) This study is in only one village (2) The previous studies still did not show any difference with the national mapping (3) The authors did not indicate any finding different with the national evidence currently used by the MoH of Ethiopia. The study area is also indicated as the high endemic area for CL on the national mapping. So, what are the base for this type of description?

Dear Reviewer, thank you for your insightful comments. Our assertion stems from several key observations: (1) Absence of National Prevalence Data for CL: The current lack of national prevalence data for Cutaneous Leishmaniasis (CL) is attributed to the outdated risk maps. These maps require validation through rapid, on-the-ground surveys, which they presently do not encompass (2) Emergence of New Locations: Recent findings indicate numerous new locations affected by CL that are not represented on existing risk maps, as highlighted in the literature (DOI:10.4269/ajtmh.20-0708). (3) Rapid Climate Change: The swift pace of global climate change suggests the possibility of new endemic sites for CL in Ethiopia. This necessitates a timely update of the risk maps to reflect these changes. In light of these factors, we have proposed a re-evaluation of the national risk areas to enable a more accurate determination.

Basically, we fear that there are many villages such as our study village, where CL is endemic but this is not reported at the national level. Hence, it remains ignored, and people in this village do not get care for CL. But we agree with your point mentioned above, that a key issue is the lack of good surveillance systems in the country, and we have stressed that now in the discussion.

- '...previously detected Leishmania parasites at the nose of one single goat (28)’: It is well explained regarding having goats at home as a risk factor. However, the authors also include the goat rearing situation in Ethiopia. It is obvious that those who are living near the gorges area commonly have goats. That might support the evidence and also give direction for future research to check the pattern more carefully.

Thanks for appreciating the finding and for the feedback.

- The 8th paragraph of the discussion part: 'A better understanding of the complex .... abundancy of infectious sand flies.': What is the importance of this paragraph for explaining this finding. It is not related to the study, and it doesn’t providing explanation based on the study ==> better to remove the whole paragraph

Dear reviewer, we see your point. However, our findings indicate that infection could not only occur away from the village, in areas where hyraxes reside, but also closer to home. To have evidence-based interventions for CL prevention, it is vital to have a good understanding about the transmission (intra/peri-domestic, remote), to understand where villagers get infected and how to intervene ideally at all levels. So we feel this is the next type of research that should be conducted. We agree it is perhaps more like a “future research needs” but think it is acceptable in a paper to – besides directly reflecting on the study findings – also to think about next steps and discuss the larger picture.

- Include the following information at the strength and limitation part of the discussion
o The study depends only in one village based on the disease distribution.

We have added this as a limitation.

o The data collectors: Physical examination was done by non-health workers

We have added this as a limitation. Nevertheless, even though the data collectors were non-health workers, with the training given, the pictures of clinical CL provided and the fact that they all are from known CL endemic areas, we believe that they were well-trained to diagnose CL cases.

o The use of sub-sample for confirmatory examination of the samples using PCR

We have added this as a limitation;

- The conclusion of the study: It is not related to the objective, method followed and finding of the study. Better to consider the finding and limitation (including the study is based on one village)

We have made some changes in the conclusion to clearly mention the key findings, besides mentioning a few recommendations coming out of our study findings.

**Reviewer #2:**

Review on “Prevalence and risk factors of cutaneous leishmaniasis (CL) in South-
Ethiopia’s newest endemic site for CL”

This is an important manuscript that described the findings of a community survey on prevalence and risk factors of cutaneous leishmaniasis (CL) in a village in southern Ethiopia where CL was previously not reported.
Authors may consider the following comments to improve the manuscript:
General
As discussed by the authors in the third paragraph of the discussion, the presence of high proportion of CL related scars indicates that the area is already endemic for CL. The area might not have been recognized as endemic due to poor surveillance and reporting system. Thus, this should not be reported as “newest” endemic site. Rather, it is important to emphasize the fact that poor surveillance and reporting system is missing to recognize such endemic sites with ongoing transmission, and thus, leaving behind such public health issues unaddressed.

We appreciate the general comment of the reviewer. Based on the comment we have adapted the title. We agree with the need for better surveillance systems and have now added this in the discussion.

On the other hand, may the low number of active CL than CL scar be a sign that CL is actually decreasing in the area?

Those with scar are mainly above the age of 12 years and is a cumulative number of cases who have had CL during their childhood. Scars remain, active lesions are usually transient. As we don’t have any previous data to determine the decline of CL in the area it is very difficult to say. The prevalence is still similar to some parts in Ethiopia. Future surveillance can tell us more about that.

Some of the independent variables considered in the risk factor analysis are not independent to each other. For example, housing conditions may be associated with income, a house with better roofing will also have better wall, outside kitchen and toilet. Similarly, the presence of animal dung in the compound will be related to keeping animals in the compound or the house. Spending outside where hyraxes reside, outside activities and spending time outside in the evening are overlapping. Analyzing suc related and overlapping variables in the same model looking for associations may lead to multicollinearity bias. Authors need to clearly explain how such possible bias was mitigated;

We agree several variables are associated, but think that is not really a problem if the association is not very strong. The purpose of multivariate analysis is to adjust for these variables in analysis. For variables that are found to be associated with the outcome in univariate analysis (eg having domestic animals), but with an association in univariate that exists only because they are associated with another variable (eg with the presence of dung), this association should disappear in multivariate analysis. For “true associations”, the association will be expected to remain (although ORs might slightly change) in multivariate analysis. The association between “holding animals” and CL was then only visible in univariate analysis due to the association with “presence of dung”, ie the effect of “dung” confounded this association. To assess for collinearity, we assessed the association in 2x2 variables and calculated the variance inflation factors. The variables “spending time outside in the evening in areas where hyraxes reside” and “type of activities outside in the evening in areas where hyraxes reside” were not included together in the multivariate analysis as all individuals having any type of activities outside in the evening in areas where hyraxes reside would automatically also have answered yes to the question “spending time outside in the evening in areas where hyraxes reside”. This would lead to strong collinearity, with the model automatically removing one variable. Instead, we conducted exploratory analysis, whereby the final model was rerun with replacing the variable ‘spending time outside late evening in areas where hyraxes live’ by the individual late evening outdoor activities, to explore which activities were associated with an increased risk of CL.

Specific comments:
Title and short title: better to avoid using the terms “newest” and “new” as explained above

Modified as suggested above.

Authors summary: The statement “The study highlights the importance of effective vector control and prevention strategies that focus on limiting human-sandfly contact in areas with hyrax presence” is generally correct statement but is not in the scope of the study findings.

We have removed this statement.

Introduction: The references mentioned in the statement, “There are however several areas in southwestern Ethiopia with similar environmental and geographical features from which CL is not reported (9,15).” to justify the absence CL case from the study area and present it as a new endemic area, do not describe the absence of CL in the study area in the past, they are CL reports from other endemic areas.

Yes, it is true. It indicates a weaker surveillance in the health system or lack of studies. Our intent is to highlight these. Our intent of that statement is that the confirmation of CL endemic sites has to come from scientific studies of such kind. We completely agree with the reviewer’s comment and have clarified that in the revised manuscript.

Methods:
- It is described that 9 out of the 25 individuals with active lesions compatible with CL were selected randomly for PCR testing of whom eight were confirmed. It is important to describe how the clinical diagnosis of CL and CL scar was made and distinguished from other similarly manifesting skin conditions.

Yes, It is mentioned in the “Conduct of the survey”, as training was given. Additional clarification is given in the revised manuscript.

- The purpose of the sensitivity analysis needs to be explained.

We have now explained this in more detail in the Methods.

Results:
- High proportion of households (86%) have indoor smoke production. This finding is not discussed in terms of its potential as insect repellent and protective means from sandfly.

Indeed, we didn’t mention much about this, as it was not found to be associated with CL in our analysis. Although there are literatures that support smoke in a house can potentially repel the vector responsible for cutaneous leishmaniasis (Youmsi et al. (2017), we did not see it in our analysis. It could potentially also depend on what kind of wood or other material is used to make smoke. In our discussion, we mainly focused on those factors associated with CL. We agree production of smoke in the house carries a risk of respiratory disease but also agree that this was not the objective of the study.

- It is strange that there are no cows and oxen owned by the study population that account 24% farmers. Oxen are common farming animals in Ethiopia.

This area has been badly affected by poverty and migration of residents to another location in search of fertile area and enough farming land. It is practically true that there are very few cows and oxen in the village.

- Eucalyptus is a common tree planted around household in Ethiopia and used for different purposes. It is thought to have some insect repellent effect. Please describe if such data was collected.

That is true. However, we do not have this kind of information.

- Table 2 shows 13 active CL and 50 CL scar in under 12 years. But in the last paragraph of the results section talking about sensitivity analysis the number is shown as 80. Please harmonize.

In this analysis, we included all active CL cases (children and adults) and CL scars upto the age of 12 (including 12 years old). We have now clarified that. Additionally Table two shows the cases < 12 years, while in analysis we used <= 12 years. We have now made this consistent across the paper.

- S2 Table: Analysis for reasons for activities outside shows 49.72% (for CL) vs 50.3% (for n CL) for fetching water/firewood with OR of 2.3 (1.6 – 3.6). It is good to revisit the analysis as such almost equal numbers may not show such statistical difference. The percentage of most of the other variables also favors no CL than CL as is discussed.

We have doubled checked this and it is correct. The reference category is “not going out” with a proportion of CL of 33.7%. Hence the risk is higher in those fetching water/fire wood (49.7%)

- Fig: S1: Indicating where the gorges and the habitats of hyrax are on the map will be more informative.

Thank you for the good suggestion. However, we find the ground coordinates for houses with CL and Scar is useful to show the distribution of cases in the village.

Discussion
- For the studies referenced (#25, #14) in the second paragraph of the discussion, clearly state if these studies have not included the current study site.

Yes, we confirm that the two references cited do not include the study village in any way. We tried to indicate that now.

- For the statement, “Several CL outbreaks have been identified over the last years, all incrementing *L aethiopica*.” please included references. (line 282),

Thanks, and references are added.

- Please consider the comments in the other sections in the discussion.

**Reviewer #3: 1. Editorial**

1.1 What does “newest endemic site” or “A new CL hotspot” mean? Does it mean the study area was not endemic for CL previously. If so, what is the evidence for that? The words “new” and “newest” are misnomers, and they should be replaced from the title and from the main body of the manuscript.

In line with a comment from reviewer 2, we have modified the title. Basically, our findings indicate the village is (long-term) endemic for CL, but this has never been investigated, reported or notified. So it is a village newly-identified to be CL endemic, although this had been missed due to the lack of a strong national surveillance system. In line with this, we have add to the need for better surveillance systems in the discussion.

2. Last sentence of the “Introduction” in page 11 begins by the phrase “Our findings aim to….”.
It makes sense to change it to “Our research (This research) aims to contribute to ……”

Thank you so much and we have corrected as suggested.

2. Regressions
2.1 Combining active CL and past CL lesions in defining a key outcome variable
Risk of CL was a key outcome variable assessed by combining active and past CL cases for the purpose of uni-(multi-) variate analysis using regression models. There were 382 cases with past lesions (390 according to table 2) whose median age was 32. Assuming most active lesions occur during childhood, the majority of the 382 cases with scars may have occurred 20 years ago. Clearly, active and past CL lesions were temporally distant from each other. Consequently, combining the active lesions with scars in formulating the outcome variable cannot be straightforward particularly that data is based on a proportionally larger of past CL lesions (scars).
For instance, S2 Table gives the univariate analysis with a breakdown by age, and shows that not only children 5 to 17, but also adults have a higher odds of CL risk (OR = 6.6 – 9.4). However, from Table 2, it was clear that children in the age bracket 5 to 11 had the highest prevalence of active CL (7.6%), and those under 5 had 1.5% prevalence, and children older than 11 years and adults had low prevalence rates (ranging from 1.0% to 2.0%). Therefore, it was clear that combining active and healed lesions was a mistake.

Thank you for your valid comment. We have given this much thought, and have acknowledged this as a limitation. The reasoning behind our approach is the following.

- Most other studies in Ethiopia have combined CL lesions and scars as outcomes, and by following this, our findings can be compared with the findings from these studies.
- Nevertheless, we also considered using only CL lesions as outcomes, but given the fact that we only had 25, and many risk factors we wanted to explore in univariable analysis. Given the rule of thumb of having 10 outcome events per factor included in univariate analysis, we felt this analysis would be underpowered and might lead to spurious associations (<https://pubmed.ncbi.nlm.nih.gov/8970487/>). Combining CL lesions and scars, would lead to a sufficient number of events. Moreover, the presence of CL scars also indicates “exposure” to CL risk “factors”, albeit more distant in time.
- However, to assess the robustness of our findings, and minimize the effect of this “exposure distant in time”, we conducted sensitivity analysis including all active CL lesions (adults and children), and all scars in children < 12 years old. As most active CL seems to occur in the age group 5-12 years, this would indicate “exposure to CL risk factors” not too long ago. In general, our findings seemed quite robust, suggesting the main model is sufficiently reliable. Nevertheless, we agree with your comment, and it is mentioned as a limitation.

2.2 Domestic animals and animal dung
Even though all households had animals, animal dung was found in 77% of households. One wonders why all household did not have dung in the compounds. Furthermore, it seems plausible to assume that presence of animals and animal dung are not independent of each other. In such a scenario, the regression model cannot assume these two variables to be independent of each other.

We think this could be related to what some villagers do with the dung. There are cultural practices whereby some residents use the dung as an organic compost for the Enset plant (false banana tree that people grow at their backyard in Gamo highlands). In the cultural practice, women might collect the dung at one place in the backyard (outside the house compound).

We agree keeping domestic animals and the presence of dung are associated, but the purpose of multivariate modeling is to adjust for variables that are associated, to see which ones, after adjusting for each other, remain associated with the outcome.

We agree several variables are associated, but think that is not really a problem if the association is not very strong. The purpose of multivariate analysis is to adjust for these variables in analysis. For variables that are found to be associated with the outcome in univariate analysis (eg having domestic animals), but with an association in univariate that exists only because they are associated with another variable (eg with the presence of dung), this association should disappear in multivariate analysis. For “true associations”, the association will be expected to remain (although ORs might slightly change) in multivariate analysis. The association between “holding animals” and CL was then only visible in univariate analysis due to the association with “presence of dung”, ie the effect of “dung” confounded this association. To assess for collinearity, we assessed the association in 2x2 variables and calculated the variance inflation factors.

In the univariate analysis, the presence of goats/sheep inside houses (but not in the compound) seemed to show marginal association with the outcome variable. Given the flaws in the design/analysis, caution should be made not to over-interpret the findings. The authors tried to build on this observation as an important finding (see discussion section implying attraction of sandflies by goats/sheep; also referring to a single natural infection of a goat - citing a publication of their own).

Valid point. However, overinterpretation was not our intention; we mainly wanted to indicate that it can’t still be ruled out and think that it merits further research. As we mentioned “Whether goat or other domestic animals … play a role as reservoir can currently not be fully ruled out and requires further work.”

In the multiple regression section, it is interesting to note that presence of domestic animals in the households did not come out as a predictor of CL incidence whereas animal dung turned out to be a remarkably significant predictive variable (univariate analysis, S3 Table).

Thank you for picking this. Yes, this was an interesting finding; as to variables that are associated, the purpose of multivariate analysis is indeed to adjust for each other and see which one remains associated with the outcome. Keeping animals and presence of dung are associated, but as mentioned above, some households with animals did not have dung around the household. As mentioned above, this could relate to variability in culture practices between the households (whereby some might collecting heaps of animal dung for compost outside of the household).

2.3 The steps involved in generating Table 3 and the adjustments made variables-wise is not clearly presented. The structure of the table is not self-explanatory. A detailed description of the model is lacking.

Thank you so much and we are sorry for the lack of clarity. We have now provided more explanation in the Methods section and in the legend of Table 3.

3. Table 1
For the section of marital status, does it make sense to re-calculate the percentages by taking the denominator as 767 (those >18 yrs)? This gives 36.5%, 49.8% and 13.7% marital status for single, married and widowed/separated respectively.

Yes, exactly. Correction is made as per your comment. Thank you!

4. Strengths and limitations
4.1 Why would the use of molecular techniques be considered as a strength? By the same token, the total population sampling seems to be related to feasibility rather than scientific justification. Apparently, the total population of the study population is small which must have incentivized the investigators to do it all without sampling. Of course, the approach minimizes sampling error.

We agree that these two points are not major strengths, so we have removed them.

The use of molecular technique was mentioned as a *relative strength* as compared to the other studies in East-Africa whereby microscopy was used to confirm CL, and microscopy has a much lower sensitivity. By using molecular method which has high sensitivity, we think our results are more reliable.

4.2 The limitations have been pointed out appropriately but remains to be a concern. As explained above, there is a deduction that older children and adults are at higher risk of acquiring CL. A close look at the data seems to point that in fact small children in the age bracket 5-11 are the ones at highest risk.

We fully agree that most active CL lesions were found in the age group 5-11 years. The CL scar data is a cumulative number of cases who have had CL, presumably mainly during their childhood. So we agree that most CL episodes occur during the age of 5-11 years. This is in line with the increase of outdoor activities at this age. By the time they reach adulthood, a lot of them will have scars, presumably acquired during their childhood.

Please also see our reply to this point above.

4.3 Because the key outcome variable was comprised of both active and healed lesions, interpretation of associations between the several presumed explanatory variables and the key outcome variable remains to be obscure, and the conclusions made are not fully substantiated.

Please see our reply to your related point above (comment 2.1). We have acknowledged this as a limitation.

5. References
5.1 Ref # 16 does not give list of authors.

Thanks, correction was done.

5.2 Journal names are given either abbreviated or written in full. Consistency is advised.

Appropriate edition was done and consistency was maintained.

6. Others
6.1 Editorial corrections will improve the quality of the manuscript.

We have made substantial editorial corrections

-
