# Supplementary material for: Prevalence and risk factors of cutaneous leishmaniasis in a newly identified endemic site in South-Ethiopia
Source: PLoS One. 2024 Dec 30;19(12):e0311917. doi: 10.1371/journal.pone.0311917 (PMC11684615; doi:10.1371/journal.pone.0311917)
Supplement: Attachment — Submitted filename: Response to Reviewers_220924.docx [file pone.0311917.s009.docx]

**Reviewer's Comments to the Author
(AUTHOR’S RESPONSES IN BLUE)**

**Reviewer #1: Data collection and entry**
Line 121: ‘… designed and uploaded to a tablet with Open Data Kit software for data collection.’
Line 159: ‘EpiData (version 2.2.3.187, EpiData Association, Odense, Denmark) was used for data entry…’
You indicated that the tools were uploaded on ODK software. Again, you also indicated the data was entered on ‘EpiData’. In your response you mentioned that ‘All data mentioned under ‘conduct of the survey’ were entered using EpiData.’ If that is the case, you have to remove that statement indicating ODK. Or, indicate which part of the data was collected using paper and ODK.

Dear reviewer, thank you for your comments. We would like to clarify that we mentioned ODK as a tool used for data collection during the survey. EpiData is listed under the statistical analysis section and is used to analyze the data collected in the field using ODK. This information is provided on line 160.

Line 230: '... of 2.5% (5% confidence interval ...' Make the correction 5% =95%

Dear reviewer, thank you and the correction is made (see Line 230 ; Page 12)

**Reviewer #3:**

In Line 281 of the manuscript, there is the following statement "Besides increasing age, spending time outside in the evening (close to where hyraxes reside) and the presence of animal dung around the house were identified as independent risk factors for CL" that hinges to the point that increasing age is a risk for CL. I have tried to point out that lumping the active and healed lesions data will be leading to scientific misinformation and misdirection of control efforts.

The authors have recognized this as a limitation and have toned down this issue overall, but retained the phrase " Besides increasing age...".

I suggest the phrase "Besides increasing age" be removed from the statement above.

Dear reviewer, we removed as you commented in the revised manuscript (Line 282, Page 15)

**Reviewer #4: Dear editor and research team,**

Thank you for sharing this work, and as advance disclaimer, I was not part of the first review round of the manuscript - so I am thankful to have the chance to look at the revision, including the comments from 3 reviewers and the answers.
The key concerns I had when reading the original manuscript, were mostly queried (namely: the far-reaching conclusion that may be too far-fetched relevant to the finding, choice of methodology of cross sectional household survey, and also on statistical analysis approaches and finding).
Few minor comments from my side that I think not yet addressed satisfactorily in the revision:
- The conclusion in the abstract, which highlight the confirmed endemicity of this one village still does not 'reflect' the benefit of this research to the community surveyed: is the village gonna be included in the national surveillance.. or there will be access to diagnosis and treatment?

Dear reviewer, thank you for mentioning this from an ethical point of view. Once reported, the village will be under regular surveillance list both by the local and national Ministry of Health. Community members were informed to use the nearest referral hospital where all CL cases are admitted and treated for free which also is the way it has been for other endemic villages.

- Related to that, the choice of this village to be exhaustively surveyed is still not clear for me- in R1 page 6 line 103- refer to wider survey of 38 villages, and the only justification to choose this is the steep valley separating it from Ochollo and also the small size of residents? Was it the case? What motivated the team to choose this one, if only just feasibility?

Dear reviewer, the article we published can give you the first work: [10.4269/ajtmh.20-0708](https://doi.org/10.4269%2Fajtmh.20-0708)

My colleague, also a PhD student, has found the sandfly responsible for transmitting CL in this village. This reinforced for our study's purpose to understand the disease burden and risk factors.

- Regarding combining CL lesion and scars- this seems indeed quite common, as in this reference (https://journals.plos.org/plosntds/article?id=10.1371/journal.pntd.0006914).
- The method stated the survey took place in 2021 but some tables refer to 2020, e.g Table 3, Table S2-S5 - which one is correct?

Dear reviewer, thank you for your attention to detail. As mentioned in the abstract and method sections, the study was conducted in 2021. Everything is correct in that respect, and we are grateful for that.

_____________________________________________________________________________________________
